# The Moderating Effect of Social Support on Parental Stress and Depression in Mothers of Children with Disabilities

**DOI:** 10.1155/2022/5162954

**Published:** 2022-03-14

**Authors:** Gyeong-A Park, Oan Na Lee

**Affiliations:** Department of Occupational Therapy, Chosun University, Gwangju, Republic of Korea

## Abstract

**Purpose:**

Mothers caring for children with disabilities often experience parental stress, which may lead them to suffer from depression. It is necessary to find a way to relieve their stress and depression. Therefore, we examined the effect of parental stress on depression and the buffering role of social support between them in a sample of 164 mothers of children with disabilities. *Participants*. One hundred and sixty-four participants (aged 25–58 years) in Gwangju and Jeollanamdo, South Korea, completed a set of self-reported measures.

**Methods:**

Parental stress, depression, and social support are assessed by Parental Stress Index, Multidimensional Scale Perceived Social Support, and Center for Epidemiological Studies Depression Scale, respectively.

**Results:**

Parental stress significantly predicted depression, and social support showed the buffering effect on the relationship between parental stress and depression among mothers of children with disabilities. These findings indicate that mothers who perceive a high level of social support are less likely to experience depression.

**Conclusions:**

This study shows the moderating effect of social support on the relationship between parental stress and depression among mothers of children with disabilities. The level of depression they experience is not that great if they perceive a high level of social support even if they experience a high level of parental stress. These findings imply that social support is a critical resource that prevents the negative effect of parental stress on depression among mothers of children with disabilities.

## 1. Introduction

Raising children brings parents joy and satisfaction. However, parents generally face a plethora of challenges after the birth of a child. Consequently, raising children induces stress and demands that the parents make numerous changes in their lifestyles and accept responsibilities [1]. Parents may experience parental stress while raising children, especially if children have disabilities [2]. For example, parents of children with oppositional behavior problems are required to care for their children more consistently than parents of children without disorders [3]. In addition, parents of children with autism spectrum disorder (ASD) reportedly experience more parental stress than parents of children without ASD or other developmental disabilities [4]. As seen in the results of previous studies, raising children with disabilities can impose burdens on the parents and families [5]. Specifically, the challenges they experience include anxiety about the future, lack of knowledge about parenting, problems with their children's jobs, social discrimination and stigma, and financial difficulties [6–8].

Also, financial and psychological burden experienced by family members of children with disabilities inevitably increases over time. When such issues remain unresolved, the tensions and conflicts within the family can become chronic and severe, even leading to breakdown of the family [9]. Family conflicts have been reported by 57.8% of families of children with disabilities, along with the finding that families experience conflict over the burden of care for children with disabilities or lack of understanding between the family members [10]. As parents raising children with disabilities require more physical, psychological, and economic resources than parents of children without disabilities [2], they experience higher levels of parental stress than parents raising children without disabilities [11].

Such a variety of burdens can cause parents of children with disabilities to experience more severe parental stress than parents of children without disabilities. The parental stress experienced by parents of children with disabilities can lead to marital conflict and divorce [4, 12, 13]. In addition to causing family dissolution by weakening the functions of the family, parental stress can also affect the psychological and physical health of parents of children with disabilities [4], and consequently, parental stress is known to be a factor that affects the psychological well-being of the caregiver [14]. Accordingly, parental stress has a negative effect on the physical and psychological health of the parents [4]. Parents of children with disabilities reportedly have a higher likelihood of suffering symptoms of depression and anxiety than parents of children without disabilities [15, 16]. Specifically, a survey on caregivers of people with developmental disabilities reported that 52% of guardians of children with disabilities received a diagnosis of suspected depression. Another study suggested that 19% of parents of children with disabilities were found to be depressed [17]. Among them, mothers who have been primary caregivers of children [18] and form strong emotional relationships with their children [19] are more impacted by their children's disabilities than any other family members. In the study of Oelofsen and Richardson [20], it was found that mothers of children with disabilities reported higher levels of parental stress than fathers. This is especially true for mothers in South Korea since they spend three times more time raising their children than fathers [21].

As previous studies indicated, parents raising children with disabilities may suffer parental stress, leading them to experience depressive symptoms [15–17, 20, 22] that impair their daily lives, reducing their quality of life [23]. Depression has a negative effect on individuals' behavior, cognition, and emotions and particularly on the individuals' quality of life [24]. Depression not only affects the daily functioning of individuals, such as changes in weight and sleep patterns, depressed mood, and loss of interest or enjoyment; it can also lead to suicidal ideation [25, 26]. Moreover, it also exacerbates conflicts over roles in the family, problem-solving ability, emotional response, and communication between family members [27]. As a result, a high level of depression of mothers with children with disabilities may have a negative impact on raising children [22]. Therefore, the depression experienced through parental stress by mothers who raise children with disabilities must be alleviated to strengthen their quality of life. Furthermore, the function of the family must be enhanced through improving the quality of life of the family members and strengthening the weakened family system.

According to the stress model of Folkman and Lazarus, the stress experienced by individuals results from the interaction between the individual and the surrounding environment. It indicates that individuals placed in an environment of deficiency or an environment that cannot satisfy their needs may experience stress [28]. Individuals' psychological vulnerability is related to the availability of psychosocial resources available to them which means that individuals' mental health can be at risk when psychosocial resources to help them adapt to stressful events are limited [29]. On the other hand, adequate psychosocial resources help individuals to cope with stress and adapt to the environment [30, 31]. Among these psychosocial resources, social support has been found to play an important role in explaining how people cope with stressful events or critical life experiences [29]. Furthermore, a buffering model suggests that social support alleviates the impact of stress on mental health [32]. Hence, social support for parents of children with disabilities could be a critical social resource that may reduce the impact of parental stress that parents of children with disabilities suffer [33].

Social support is an important coping resource for parents of children with disabilities, as it also plays a role in relieving stress [34]. Social support refers to individuals' subjective evaluation that they will be able to receive the desired support and help at the time of need from the surrounding social networks including their spouse, relatives, friends, coworkers, or a community [35], which benefits the individuals' physical and psychological health [36]. According to the buffering model, social support is the moderator that reduces the negative emotions caused by stressful events [32]. Hence, it is known as a coping resource to cope with stress and adapt to the surrounding environments [37]. Specifically, individuals who experience stress experienced less depression if social support they perceived was adequate [38–40]. Therefore, even if parents of children with disabilities experience parental stress, if their perceived social support is abundant, their negative emotions caused by parental stress could be reduced.

However, there is a dearth of studies focusing the buffering effect of social support of parental stress on depression of mother of children with disabilities in South Korea. There is a study that examined the relationship among parental stress, depression, and social support of mothers of children with disabilities, but participants of the study were in Seoul, South Korea [41]. As the capital of Korea where the central government is located, Seoul has a large gap with other regions because it has abundant resources compared to other regions, especially Gwangju and Jeollanamdo. Therefore, this study is aimed at examining the role of informal social support from families, friends, and social networks in the community in the relationship between parental stress and depression among mothers of children with disabilities residing in Gwangju and Jeollanamdo, South Korea, and discussing social interventions accordingly.

## 2. Materials and Methods

### 2.1. Procedures

Upon receiving approval from the Institutional Review Board of Chosun University (2-104105-AB-N-01-2020-43), participants were recruited by flyers posted at special education schools, rehabilitation centers, and clinics in Gwangju and Jeollanamdo, South Korea. Mothers of children with disabilities who agreed to participate were provided a package consisting of a written informed consent form, a demographic questionnaire, Parental Stress Index-Short Form (PSI-SF), Social Support (MSPSS), and Depression Scale (CES-D).

### 2.2. Participants

A sample size calculation using G∗Power resulted in a required minimum sample size of 107 (effect size = .15, *α* = .05, 1-*β* = .95). To allow for incomplete data, we recruited more than the required minimum sample size. Our study included 164 mothers of children with disabilities in Gwangju and Jeollanamdo, South Korea, and ages of participants ranging from 25 to 58 years (*M* = 40.8, SD = 6.3) with the monthly income ranging from 0 to 8,000,000 won (*M* = 2,802,195.12, SD = 1,423,321.98).

The characteristics of the children were as follows. Of those children with disabilities, 98 (59.8%) were male, and 66 (40.2%) were female. Children's ages ranged from 4 to 20 years (*M* = 10.6, SD = 4.3). Among children, 19 (11.6%) were diagnosed with visual/hearing/language disability; 72 (43.9%) were diagnosed with intellectual disability; 8 (4.9%) were diagnosed with physical disability; and 65 (39.6%) were diagnosed with autism spectrum. Detailed characteristics of participants are presented in Tables 1 and 2.

### 2.3. Measures

#### 2.3.1. Parental Stress Index

Parental stress was assessed by the Korean version of the Parental Stress Index-Short Form (K-PSI-SF) [42]. It is a 36-item self-report scale designed to assess the degree of distress resulting from child-rearing. An example of an item is “Gave up my life for children's needs.” Each item is rated on a 5-point Likert scale ranging from 1 (strongly disagree) to 5 (strongly agree). Total scores can range from 36 to 180, and higher scores indicate a higher level of distress from child-rearing. The coefficient alpha in the study by Lee and colleagues [42] was .91, and the coefficient alpha in the present study was .92.

#### 2.3.2. Social Support

Perceived social support was assessed by the Korean version of the Multidimensional Scale of Perceived Social Support (MSPSS; [43]). It is a 12-item self-report scale designed to assess the degree of social support from family members, friends, and informal social networks in the community that participants perceive. An example of an item is “There is a special person who is around when I am in need.” Each item is rated on a 7-point Likert scale ranging from 1 (very strongly disagree) to 7 (very strongly agree). Total scores can range from 12 to 84, and higher scores indicate a higher level of perceived social support. The coefficient alpha in Zimet and colleagues [43] was .88. The coefficient alpha in the present study was .90.

#### 2.3.3. Depression

Depressive symptoms were assessed by the Korean version of the Center for Epidemiological Studies Depression Scale (CES-D; [44]). It is a 20-item self-report scale designed to assess the level of depressive symptoms. An example of an item is “My sleep was restless,” and each item is rated on a 4-point Likert scale ranging from 0 (rarely) to 3 (most of the time). Total scores can range from 0 to 60, and higher scores indicate higher levels of depressive symptoms. CES-D scores of 16 or higher indicate a possible risk of clinical depression [45]. The coefficient alpha for the general population was .85 and 90 for a psychiatric population [46], and the coefficient alpha in the present study was .90.

### 2.4. Data Analysis

To examine the moderating effect of social support on the relationship between parental stress and depression, the predictor and moderator variables, parental stress, and social support, respectively, were standardized before computing the interaction term to reduce multicollinearity which is recommended by Frazier et al. [47] for moderation analysis. To standardize variables, the mean was subtracted for each observation and divided by standard deviation. Then, a hierarchical multiple regression analysis was conducted. For hierarchical multiple regression, demographic variables (age, income, and education level) were entered as controlled variables in step 1. Then, parental stress and social support variables were entered to examine the main effects of these variables in step 2. Finally, parental stress, social support, and the interaction term computed through the multiplication of standardized parental stress and social support were entered in step 3. The increment in *R*^2^ (Δ*R*^2^) for step 3 indicates the moderating effect of social support in the relationship between parental stress and depression [48]. Statistical significance was set at *p* < .05. To further explore the interaction effect of parental stress and social support on depression graphically, the two-way interaction graph was plotted.

## 3. Results

### 3.1. Descriptive Statistics and Correlations for the Measured Variables

Descriptive statistics and correlations for measured variables are presented in Table 3. The mean scores of parental stress, social support, and depression were 105.11 (SD = 21.47, range = 52.50–172.50), 35.91 (SD = 8.36, range = 12.00–52.00), and 19.52 (SD = 11.48, range = 2.00–54.00), respectively. A total of 89 (54.3%) participants scored 16 or higher on the CES-D in the present study. For correlations, parental stress was positively correlated with depression (*r* = .70, *p* < .001) and inversely correlated with social support (*r* = −.49, *p* < .001).

### 3.2. Main Effects of Parental Stress and Social Support on Depression

The main effects of parental stress and social support on depression are presented in step 2 (Table 4). Parental stress and social support accounted for 63.0% of the variance in depression. Specifically, parental stress (*β* = .54, *p* < .001) and social support (*β* = −.33, *p* < .001) were significant predictors of depression (*R*^2^ = .63, *p* < .001).

### 3.3. Moderating Effect of Social Support on Parental Stress and Depression

**Figure 1:**
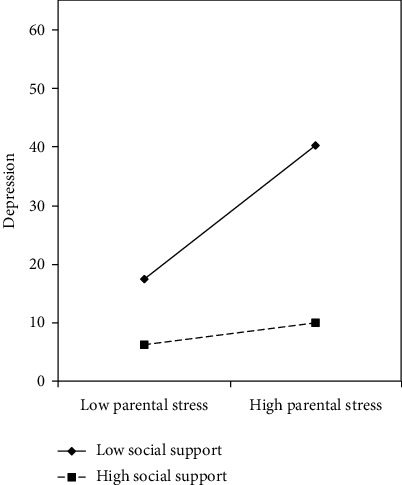
The moderating effect of social support.

In step 3 (Table 4), two-way interaction (parental stress × social support) significantly predicted depression (*β* = −.23, *p* <.05). Specifically, the two-way interaction term accounted for an additional 4.0% (Δ*R*^2^ = .04) of the variance in depressive symptoms. This indicates the moderating effect of social support on parental stress and depression. To further explore the interaction effect of parental stress and social support on depression graphically, the two-way interaction effect was plotted. As presented in Figure 1, when parental stress increased, parents of children with disabilities who perceived low level of social support to cope with parental stress were vulnerable to depression. Conversely, those who perceived high level of social support to cope with parental stress were less vulnerable to depression.

## 4. Discussion and Conclusion

The purpose of the present study is to examine the effect of parental stress on depression and the buffering effect of social support between parental stress and depression among mothers of children with disabilities. Therefore, we examined the effect of parental stress on depression and the moderating effect of social support between them in a sample of 164 mothers of children with disabilities in Gwangju and Jeollanamdo, South Korea. The results and the discussion are as follows.

The present study confirms the association between parental stress, depression, and social support, which is consistent with previous studies [16, 33]. Specifically, there is a significantly positive correlation between parental stress and depression, which means that a high level of parental stress is associated with a high level of depression. Also, social support is negatively correlated with parental stress and depression which means that a low level of social support is associated with a high level of parental stress and depression. Mothers of children with disabilities who perceive a low level of social support experience high levels of parental stress and depression. These results are consistent with previous studies that show when social support is limited, the level of stress and depression was high [37–40].

Furthermore, the regression analysis shows the effect of parental stress on depression, which finding is consistent with previous studies [4, 14], and confirms the adverse effect of taking care of children with disabilities on mothers' mental health. Specifically, mothers who experience parental stress taking care of their children with disabilities may suffer from a high level of depression. Given the adverse effect of parental stress on mothers of children with disabilities, interventions and programs that reduce parental stress may prevent mothers of children with disabilities from suffering depressive symptoms.

Lastly, social support from extended family members, friends, and informal social networks in the community moderates the influence of parental stress on depression among mothers of children with disabilities. This indicates that a high level of informal social support weakens the negative effect of parental stress on depression. Mothers of children with disabilities who experience a high level of parental stress can suffer from a high level of depression. However, if they are provided with adequate informal social support from extended family members, friends, and informal social networks in the community, their depressive symptoms may be reduced. On the other hand, those mothers with a low level of informal social support can suffer from depressive symptoms due to parental stress. Consistent with previous studies, the present study confirms that social support is the coping resource of stress that alleviates the effect of stress on individuals' mental health [4, 31, 33].

The findings of the present study indicate that providing informal social support can be a great coping resource to alleviate the depression caused by parental stress. Raising children with disabilities is associated with many difficulties that impair mothers' psychological well-being. The stress experienced during the nurturing of children with disabilities is a factor that threatens the psychological well-being of mothers of children with disabilities [14]. However, although high parental stress contributes to depression among mothers of children with disabilities, the results of this study identify that the level of depression decreases when the mothers' perceived level of informal social support around them is high. Families of children with disabilities in South Korea are reluctant to access the public social support due to the stigmatization and prejudice toward disability [49]. For this reason, as shown in the results of this study, mothers of children with disabilities seem to prefer informal social support.

As obstacles to their psychological well-being, mothers of children with disabilities reported difficulties in accessing social services and information, financial difficulties, and difficulties integrating into schools and communities while raising their children [6]. According to a study that examined their needs, mothers of children with disabilities reported need of services for financial and care support, increased support for treatment and education services, vocational training for their children, and transitional programs such as enhancing social skill programs [10]. In order to prevent parental stress-induced depressive symptoms among mothers of children with disabilities, their parental stress and depressive symptoms must be alleviated through provision of appropriate social services. Specifically, integration of informal social support and public social service is needed. For example, forming a support group with mothers of children with disabilities in the community to encourage each other and exchange information can help ease their stress and support them.

Furthermore, providing programs for families to participate would alleviate family conflicts and strengthen the functioning of the family since families of children with disabilities experience conflict over the burden of caring children with disabilities. In previous studies, active and cooperative parenting participation of mothers and fathers is treated as an important factor in lowering parenting stress [50]. However, fathers in South Korea generally spend less time parenting children than mothers, and especially, fathers with a traditional value of gender role were found to be less involved in parenting children [21]. Therefore, programs should be provided for fathers that facilitate their participation in parenting, meetings and family camps organized for siblings of children with disabilities, promotion of parental groups and activation of self-help groups, and the operation of a resource centers to provide systematic parental education and training support for the parents. At the same time, educations of the general community through mass media are needed to reduce stigma and prejudice associated with disability. Families in South Korea are reluctant to seek public social support due to stigmatization and prejudice toward disability [49]. Also, because saving face of family is so important in Korea [51], families of children with disabilities may not seek public social support. Therefore, it is necessary to provide educations for general populations to reduce stigma and prejudice associated with disability so that families of children with disabilities can seek public social support without worrying about social stigma and prejudice toward disability.

If the negative emotions of family members remain unresolved, tensions and conflicts within the family can become chronic and severe, which can even lead to family destruction [9]. Parental stress experienced by mothers of children with disabilities can impair their psychological well-being and have an impact on their overall family system [16]. Therefore, effective and efficient informal and formal social support must be provided to the mothers of children with disabilities to protect them from psychological difficulties that may occur while they raise their children.

Although this study highlights the importance of social support in the family of children with disabilities, there are a few limitations. First, as the study was cross-sectional in design and the participants of the study are limited to mothers of children with disabilities in Gwangju and Jeollanamdo, South Korea, caution must be taken when generalizing the results of this study. Second, there are many different types of social support such as emotional, economic, informational, and instrumental support that mothers of children with disabilities can obtain. However, this study only considered informal social support from extended families, friends, and informal social networks in the community. Therefore, it is difficult to determine which type of social support more effectively reduces depression caused by parental stress among mothers of children with disabilities. Future studies thus need to investigate the effects of other types of social support. Lastly, this study did not explore the parental stress according to the types of disability. Parental stress experienced by mothers of children with disabilities may vary according to the types of disability, but this study did not examine the parenting stress according to the types of disability. Therefore, future studies must explore parental stress according to the types of disability and identify the types of disability that cause the highest level of parental stress and depression among mothers of children with disabilities.

## Figures and Tables

**Table 1:** General characteristics of mothers of children with disability (*N* = 164).

Classification	Number (*N*)	Percent (%)
Age		
20~29	7	4.3
30~39	63	38.4
40~49	82	50.0
50~59	12	7.3
Academic education background		
High school graduate	58	35.4
Two-year college graduate	35	21.3
University or higher	71	43.3
Monthly income (₩)		
Less than 1,000,000	14	8.5
1,000,000~2,000,000	38	23.2
2,000,000~3,000,000	45	27.4
More than 3,000,000	67	40.9

**Table 2:** General characteristics of children with disability (*N* = 164).

Classification	Number (*N*)	Percent (%)
Sex		
Male	98	59.8
Female	66	40.2
Age		
1~5	18	11.0
6~10	81	49.4
11~15	30	18.3
16~20	35	21.3
Type of disability		
Visual/hearing/language disability	19	11.6
Intellectual disability	72	43.9
Physical disability	8	4.9
Autism spectrum	65	39.6
Period of disability		
Less than a year	32	19.5
1 to 4 years	71	43.3
More than 4 years	61	37.2

**Table 3:** Correlation and descriptive statistics for measured variables (*N* = 164).

Variables	1	2	3	*M*	SD	Range
(1) Parental stress	1			105.11	21.47	52.50-172.50
(2) Social support	-.49^∗∗∗^	1		35.91	8.36	12.00-52.00
(3) Depression	.70^∗∗∗^	-.64^∗∗∗^	1	19.52	11.48	2.00-54.00

^∗∗∗^ *p* < 0.001.

**Table 4:** Hierarchical multiple regression analyses testing moderating effect of social support in the relationship of parental stress and depression.

Predictor	*B*	SE	*β*	*R* ^2^	Δ*R*^2^	Δ*F*
Step 1						
Intercept	7.69	6.02				
Age	.20	.14	.11			
Income (￦)	-.00	.00	-.03	.09^∗∗^	.09^∗∗^	3.74^∗∗^
Education level^a^						
College	4.74	2.41	.17			
University or	7.19	2.15	.31^∗∗^			
higher			-			
Step 2						
Intercept	14.31	3.98		.63^∗∗∗^	.54^∗∗∗^	113.32^∗∗∗^
Parental stress	.29	.03	.54^∗∗∗^			
Social support	-.45	.09	-.33^∗∗∗^			
Step 3						
Intercept	16.60	3.78				
Parental stress	.26	.03	.49^∗∗∗^	.67^∗∗∗^	.04^∗∗∗^	20.96^∗∗^
Social support	-.53	.08	-.38^∗∗∗^			
Interaction	-.01	.00	-.23^∗^			

Interaction: interaction of parental stress and social support. ^a^Dummy coding. ^∗∗^*p* < .01 and^∗∗∗^*p* < .001.

## Data Availability

The data used to support the findings of this study are available from the corresponding author upon request.
